# Shortcut learning leads to sex bias in deep learning models for photoacoustic tomography

**DOI:** 10.1007/s11548-025-03370-9

**Published:** 2025-05-09

**Authors:** Marcel Knopp, Christoph J. Bender, Niklas Holzwarth, Yi Li, Julius Kempf, Milenko Caranovic, Ferdinand Knieling, Werner Lang, Ulrich Rother, Alexander Seitel, Lena Maier-Hein, Kris K. Dreher

**Affiliations:** 1 Division of Intelligent Medical Systems (IMSY), German Cancer Research Center (DKFZ), Heidelberg, Germany; 2 Faculty of Mathematics and Computer Science, Heidelberg University, Heidelberg, Germany; 3 Medical Faculty, Heidelberg University, Heidelberg, Germany; 4 Department of Vascular Surgery, University Hospital Erlangen, Friedrich-Alexander-Universität Erlangen-Nürnberg (FAU), Erlangen, Germany; 5 Department of Pediatrics and Adolescent Medicine, University Hospital Erlangen, FAU, Erlangen, Germany; 6 National Center for Tumor Diseases (NCT), NCT Heidelberg, a Partnership Between DKFZ and University Hospital Heidelberg, Heidelberg, Germany; 7 Faculty of Physics and Astronomy, Heidelberg University, Heidelberg, Germany

**Keywords:** Sex Bias in AI, Shortcut learning, Photoacoustic tomography (PAT), Peripheral artery disease (PAD)

## Abstract

**Purpose:**

Shortcut learning has been identified as a source of algorithmic unfairness in medical imaging artificial intelligence (AI), but its impact on photoacoustic tomography (PAT), particularly concerning sex bias, remains underexplored. This study investigates this issue using peripheral artery disease (PAD) diagnosis as a specific clinical application.

**Methods:**

To examine the potential for sex bias due to shortcut learning in convolutional neural network (CNNs) and assess how such biases might affect diagnostic predictions, we created training and test datasets with varying PAD prevalence between sexes. Using these datasets, we explored (1) whether CNNs can classify the sex from imaging data, (2) how sex-specific prevalence shifts impact PAD diagnosis performance and underdiagnosis disparity between sexes, and (3) how similarly CNNs encode sex and PAD features.

**Results:**

Our study with 147 individuals demonstrates that CNNs can classify the sex from calf muscle PAT images, achieving an AUROC of 0.75. For PAD diagnosis, models trained on data with imbalanced sex-specific disease prevalence experienced significant performance drops (up to 0.21 AUROC) when applied to balanced test sets. Additionally, greater imbalances in sex-specific prevalence within the training data exacerbated underdiagnosis disparities between sexes. Finally, we identify evidence of shortcut learning by demonstrating the effective reuse of learned feature representations between PAD diagnosis and sex classification tasks.

**Conclusion:**

CNN-based models trained on PAT data may engage in shortcut learning by leveraging sex-related features, leading to biased and unreliable diagnostic predictions. Addressing demographic-specific prevalence imbalances and preventing shortcut learning is critical for developing models in the medical field that are both accurate and equitable across diverse patient populations.

**Supplementary Information:**

The online version contains supplementary material available at 10.1007/s11548-025-03370-9.

## Introduction

Convolutional neural networks (CNNs) are widely used for medical image analysis but can exhibit demographic bias in their predictions leading to performance disparities across demographic subgroups [1]. One potential cause of these disparities is shortcut learning, where models learn spurious correlations or shortcuts, resulting in unreliable predictions.

While shortcut learning has been studied in common medical imaging domains, such as X-ray imaging, computed tomography (CT), and magnetic resonance (MR) imaging [1–5], it remains largely unexplored in emerging modalities, such as PAT. PAT is a non-ionizing interventional imaging modality that combines the high contrast of optical imaging with the high resolution of ultrasound (US) imaging [6]. Compared to US imaging, which uses a sound-in sound-out principle, PAT is based on a light-in sound-out principle. Using multiple wavelengths, PAT can resolve functional tissue properties such as oxygen saturation in real time [7]. Existing PAT systems—such as the CE-certified MSOT Acuity Echo used in this study—are often hybrid imaging systems that allow for a joint acquisition of US and PAT images in real time essentially enabling a combined structural and functional interventional imaging several cms deep in the tissue. While PAT as an interventional imaging modality is still emerging, it has already been proven to be an asset in various interventional settings for photoacoustic-guided hysterectomy [8], needle tracking [9], interventional guidance in cardiovascular medicine [10], and surgery [11], as well as first applications in the context of da Vinci robotic interventions [12]. The use of deep learning in PAT has especially been increasingly studied [13]. However, the topic of shortcut learning leading to sex bias in PAT has received no attention to date, despite the awareness of sex differences in the field of PAT [14]. So far, the only source of bias that has received substantial attention in the literature in the context of PAT is skin tone [15], as different skin tones interact differently with light [16]. Previous studies with other medical image modalities, such as X-ray imaging have shown that sex-specific prevalence imbalances can lead to subgroup performance disparities [17]. The severity of the impact that subgroup separability can have, however, heavily varies between medical imaging modalities [3], thus highlighting the importance of investigating this issue for each modality separately. To our knowledge, there is no literature on the impact of sex bias in deep learning models for PAT to date. The purpose of this work was therefore to shed light on this important issue, using PAD diagnosis as a representative sample clinical application. PAD is particularly suitable for this investigation due to the strong causal influence of sex on its expression [18].

PAD is a prevalent circulatory condition where narrowed arteries reduce blood flow to the limbs. Early and accurate diagnosis of PAD is essential to prevent serious complications like limb amputation. CNN-based support in the current clinical workflow for PAD diagnosis with PAT can help automate and accelerate initial examinations, facilitating earlier diagnosis.

Given the gap in the scientific literature concerning biases in PAT in general and in PAD diagnosis in particular, our main contributions are threefold: (1) We are the first to show that neural networks can predict sex from PAT images, indicating that sex-specific features are present in PAT data. (2) Using PAD diagnosis as an example, we demonstrate that models trained on datasets with imbalanced sex-specific prevalence ratio (PRs) exhibit significant performance degradation when tested on balanced datasets and display severe underdiagnosis disparity between sexes, particularly affecting the underrepresented sex. (3) We provide evidence that neural networks trained for PAD diagnosis encode sex-related features, as demonstrated by effective reuse of learned feature representations between PAD diagnosis and sex classification tasks.

## Materials and methods

**Fig. 1:**
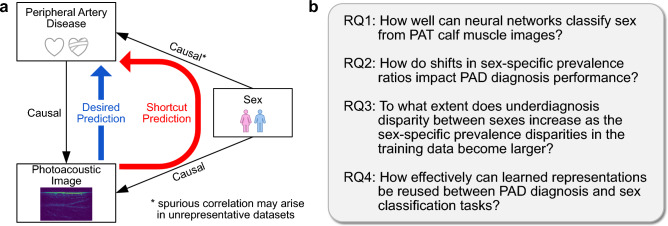
Summary of contribution **a** Illustration of shortcut learning in the context of peripheral artery disease (PAD). **b** Specific research questions (RQs). PAD induces vascular changes that causally influence photoacoustic tomography (PAT) images, allowing for automatic diagnosis. However, sex also affects PAT images, influenced by factors such as differences in skin and fat layer thickness. While sex might have a causal link to the risk and presentation of PAD, some datasets might exhibit an overemphasized spurious correlation between PAD and sex. In this case, neural networks may over-rely on the sex, i.e. inadvertently using it as a shortcut for PAD prediction, leading to biased models

This work is based on the hypothesis that CNN-based models trained on PAT data can exhibit sex bias due to shortcut learning, impacting the reliability and fairness of neural networks. To investigate this hypothesis, this work addresses the research questions (RQs) depicted in Fig. 1.

**Table:** 

Terminology	
*Sex* The biological attributes distinguishing males and females.	
*Sex bias* The improper influence of sex-related features in a model’s predictions, where these features serve as non-causal proxies for the factors directly related to the target prediction.	
*Shortcut learning* The phenomenon where a machine learning model relies on spurious correlations or easily learned features not directly causal to the target variable [2]. In our context, models may exploit sex-related features as shortcuts for disease prediction (depicted in Fig.1).	
*Sex-specific PR* The imbalance of disease prevalence between sexes in a dataset, defined as the proportion of diseased males over the proportion of diseased females. Furthermore, sex-specific prevalence shifts refer to changes in the PR between datasets (e.g., between training and test sets).	

*Dataset and data sampling strategy*

The CE-certified MSOT Acuity Echo (iThera Medical GmbH, Munich, Germany) was used to capture 2D photoacoustic images of the calf muscle from 147 individuals at wavelengths 760, 800, and 850 nm. Data was acquired within two clinical studies at the Department of Vascular Surgery, University Hospital Erlangen, Germany under study IDs NCT05373927 and NCT05773534. The health status was diagnosed by PAD experts using established methods, including angiographic imaging, and was characterized by intermittent claudication of mild to moderate severity (Fontaine IIa/IIb) [19].

**Table 1:** Sample count distribution by sex-specific prevalence ratio (PR)

	Training set (N = 48)				Validation set (N = 12)				Test set (N = 24)			
PR	F-H	F-D	M-H	M-D	F-H	F-D	M-H	M-D	F-H	F-D	M-H	M-D
1	12	12	12	12	3	3	3	3	6	6	6	6
2	16	8	8	16	4	2	2	4	8	4	4	8
5	20	4	4	20	5	1	1	5	10	2	2	10
$\infty$	24	0	0	24	6	0	0	6	12	0	0	12

F represents female, M represents male, H represents healthy, and D represents diseased

The data was divided into three pools: a training pool ($\text {N}_\text {train}$ = 86), a validation pool ($\text {N}_\text {val}$ = 21), and a test pool ($\text {N}_\text {test}$ = 40). These splits remained fixed throughout all experiments and were created using confounder matching, specifically controlling for sex and disease status, to ensure that the sex-disease strata were evenly distributed across all data splits. The data pools were subsequently used to randomly sample the desired sex-specific PR. Importantly, across all experiments, the PR in the validation sets was kept consistent with that of the training sets. Table 1 presents the sampling counts for each sex-disease stratum at each PR. In all of our experiments, the total number of male and female patients, as well as the total number of healthy and diseased patients, were consistently balanced. The key variable manipulated across experiments was the distribution of diseased patients between sexes to achieve the desired sex-specific PR.

*Classification model*

A version of EfficientNetV2_B0 [20], pre-trained on ImageNet [21], was used for the classification tasks in all experiments. Cross-entropy loss was employed as the loss function. Hyperparameter optimization was conducted manually only once for the PAD classifier trained on the balanced dataset (PR = 1) using the corresponding validation set. The optimized hyperparameters were then applied unchanged to all other models, including the one for sex classification and those trained on datasets with different sex-specific PRs. Details on data processing and hyperparameters are provided in the supplementary material.

*Experimental design*

In order to answer the RQs posed in Fig. 1, four corresponding experiments were designed. For each classifier in these experiments, an ensemble of 10 models was trained. Each model in the ensemble was trained using a unique randomized sampling from the training and validation pools, maintaining the predefined sex-specific PR. Sample counts per sex-disease group for each PR are provided in Table 1. For performance evaluation, AUROC was used following recommendations of [22]. Each ensemble for RQs 1, 2, and 4 (not used for RQ3) was evaluated using stratified bootstrapping ($\text {n}_\text {iter}$ = 1000) to generate 95 % confidence intervals (CIs) for the reported AUROC scores. Reporting confidence intervals is essential, as recent studies have shown that the performance variability in medical image analysis models can be substantial. For instance, Christodoulou et al. [23] found that the median width of confidence intervals for MICCAI 2023 segmentation models was three times larger than the median performance gain over previous methods, highlighting the importance of variability analysis in model evaluation. In each iteration, the test pool was sampled with replacement according to the predefined PR. The sample counts per stratum for each PR are provided in Table 1.

*RQ1, Sex classification in PAT* The experiments are divided in:

1. Sex separability in PAT: A sex classifier was trained and tested using the balanced sex-disease distribution (PR = 1). The result was compared to a PAD classifier which was trained and tested under the same conditions.
2. Generalizability to other datasets: The sex classifier was applied as-is to a dataset of 525 PAT images that were acquired at three body sites of 30 healthy volunteers: calf, forearm, and neck (see S2.2 for further details).

*RQ2, Impact of sex-specific PR shifts*

PAD classifiers were trained on four different distributions with sex-specific (PRs) of 1, 2, 5, and $\infty$ (where all diseased individuals were male). Each ensemble was evaluated on test sets with each of the four different PRs.

*RQ3, Underdiagnosis disparity*

PAD classifiers were trained on four different distributions with sex-specific PR of 1, 2, 5, and $\infty$. We report the mean and median underdiagnosis disparity [24] between females and males across 10 separate runs. Underdiagnosis disparity is defined as the difference in the ratio of false negatives to false positives between females and males. A positive value indicates an underdiagnosis bias against females. For each run, a new ensemble was trained and the whole test pool ($\text {N}_\text {test}$=40) was used to calculate the underdiagnosis disparity, which measures the difference in diagnostic accuracy between sexes.

*RQ4, Feature representation similarity* The experiments are divided in:

1. Transfer learning for sex and PAD classification: This experiment investigated whether CNNs reuse learned representations between PAD diagnosis and sex classification. To achieve this, the feature extractors of the sex and PAD classifiers from RQ1 were frozen, and the classification head was retrained on the alternate task (PR = 1 for all datasets).
2. Principal component analysis (PCA) projections: This experiment aimed to analyze and visualize the feature representations of PAD classifiers trained on balanced (PR = 1) and extremely imbalanced (PR = $\infty$) datasets using PCA. By assessing the distribution differences of the feature representations with respect to sex and disease status using 2-dimensional Wasserstein distances calculated on the first two principal components, additional insights can be drawn whether models trained on imbalanced data encode sex-related features more prominently. For PR = 1 and PR = $\infty$, a representative model was chosen from the corresponding ensemble that showed median AUROC performance on the in-distribution test set within the ensemble. We randomly drew 7 samples for each sex-disease subgroup from the test pool, ensuring a test set with PR = 1, resulting in 28 samples in total. To avoid sampling bias, we performed 1000 sampling runs, and then calculated descriptive statistics of the Wasserstein distance over all runs.

## Results

This section presents the results of the experiments described in the previous section corresponding to the four driving RQs of this study.

*RQ1, Sex classification in PAT*

**Fig. 2:**
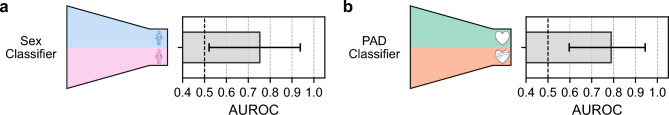
Sex separability **a** and peripheral artery disease (PAD) separability **b** are possible based on photoacoustic tomography images of the calf muscle. The mean area under the receiver operating characteristic curve (AUROC) is shown for sex **a** and PAD **b** classification models that were trained and tested on balanced data (sex-specific prevalence ratio PR = 1). Whiskers indicate the 95 % confidence intervals, and the dashed black lines at AUROC = 0.5 mark the performance expected from random guessing

**Fig. 3:**
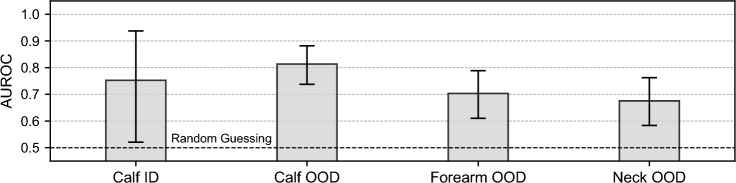
A sex classifier trained on a peripheral artery disease (PAD) dataset (PR = 1) generalizes well to out-of-distribution (OOD) datasets of healthy volunteers across three body sites (calf, forearm, and neck). The sex classifier was tested on both the in-distribution (ID) test set (PR = 1) and OOD test sets. The mean area under the receiver operating characteristic curve (AUROC) is shown as a dot, and whiskers represent 95 % confidence intervals

1. Sex separability in PAT: As shown in Fig. 2, the sex classifier was able to classify the subject’s sex from calf muscle PAT images, achieving a performance of 0.75 (95 % CI: 0.52–0.94). This is comparable to its performance in diagnosing PAD (0.79, 95 % CI: 0.60–0.94).
2. Generalizability to other datasets: As can be seen in Fig. 3, the sex classifier trained on the PAD dataset and tested on a healthy volunteer dataset scored mean AUROC results of 0.81 (95 % CI: 0.74–0.88) for the calf, 0.70 (95 % CI: 0.62–0.79) for the forearm, and 0.68 (95 % CI: 0.59–0.77) for the neck.

*RQ2, Impact of sex-specific PR shifts*

**Fig. 4:**
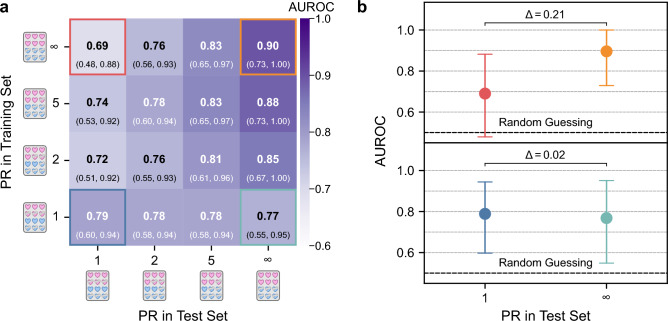
Impact of sex-specific prevalence shifts from training set to test set. Peripheral artery disease classifiers trained on distributions with increasing sex-specific prevalence ratios (top to bottom PR = $\infty$, 5, 2, 1) show significant performance drops and instability when tested on balanced data (PR = 1). Performance is measured with the mean area under the receiver operating characteristic curve (AUROC) shown in bold **a** and as a dot **b**. The model trained with PR = $\infty$ experienced a 0.21 AUROC drop. Brackets **a** and whiskers **b** represent 95 % confidence intervals

The model trained with a balanced distribution (PR = 1) maintained consistent performance across all test domains (cf. Fig. 4a), demonstrating robustness to shifts in sex-health distributions. The model trained with PR = $\infty$ (all diseased individuals male) showed a 0.21 drop in AUROC when tested on a balanced test domain (PR = 1) (cf. Fig. 4b, revealing a strong performance degradation when facing domain shifts. Increased sex-specific prevalence bias during training led not only to less stable outcomes but also to an overestimation of performance when models were tested on datasets with similarly high PRs.

*RQ3, Underdiagnosis disparity*

**Fig. 5:**
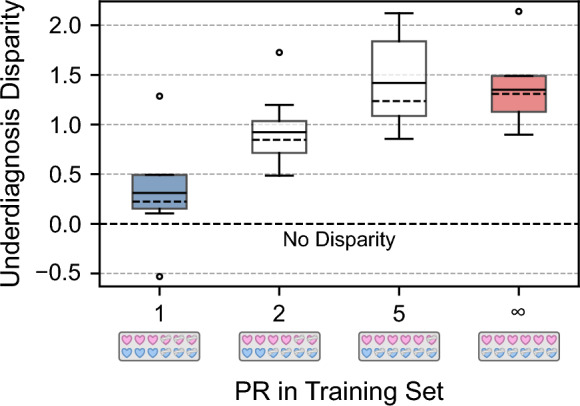
The underdiagnosis disparity between sexes increases with higher sex-specific prevalence ratio (PR) in the training data. The solid lines represent the mean and the dashed lines represent the median underdiagnosis disparity across ten runs of trained ensembles. The boxes show the interquartile range (IQR), while the whiskers extend to 1.5 times the IQR from the box

Both the mean and median underdiagnosis disparity between sexes generally increase as the sex-specific PR in the training data increases (cf. Fig. 5). Although the median underdiagnosis disparity increases from PR = 5 to PR = $\infty$, the mean slightly decreases. However, both values fall within each other’s interquartile range (IQR).

*RQ4, Feature representation similarity*

**Fig. 6:**
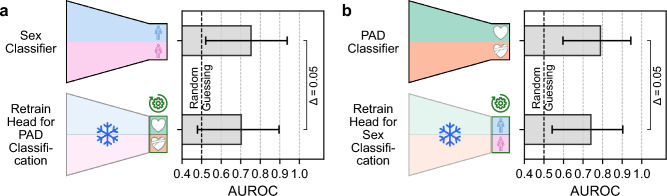
Transfer Learning is possible between sex and peripheral artery disease (PAD) classification encodings. The last layers (heads) of the sex and PAD classifiers from RQ1 (cf. Fig. 2) indicated by a green gear symbol, were retrained for the opposite task. Retraining and testing were conducted on balanced data (sex-specific prevalence ratio PR = 1). Snowflakes indicate frozen layers. The mean area under the receiver operating characteristic curve (AUROC) is shown for sex **a** and PAD **b** classification models. Whiskers indicate the 95 % confidence intervals

**Fig. 7:**
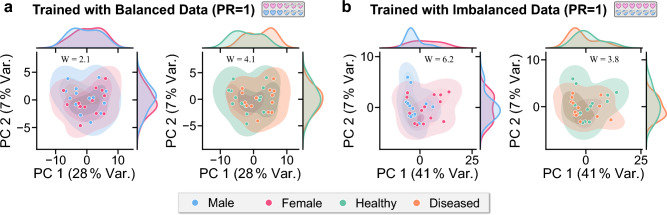
Learned representations of peripheral artery disease (PAD) classifiers exhibit stronger sex separation in the principal component analysis (PCA) projections when trained with extreme sex-specific prevalence ratios (PR = $\infty$) compared to those trained on balanced PR data (PR = 1). The first two PCA components of the learned representations of a balanced subset of the test pool and the distributions of the marginals are displayed for models trained with PR = 1 **a** and PR = $\infty$ **b**, with the variance explained indicated in brackets in the axis labels. W indicates the 2D-Wasserstein distance between the subgroups

1. Transfer learning for sex and PAD classification: The sex classifier retrained for PAD classification experienced a drop in AUROC of 0.05 from 0.75 (95 % CI: 0.52– 0.94) to 0.70 (95 % CI: 0.48–0.90) (cf. Fig. 6). The PAD classifier retrained for sex classification experienced a similar drop in AUROC of 0.05 from 0.79 (95 % CI: 0.60–0.94) to 0.74 (95 % CI: 0.54–0.90) (cf. Fig. 6). Both retrained classifiers, however, still perform substantially better than random guessing.
2. PCA projections: The PCA results achieved from a representative subset, selected as the subset with Wasserstein distances closest to the geometrical median calculated across 1000 sampling runs, are shown in Fig. 7. The first two principal components of the PR = $\infty$ model show higher differences in the distributions between the sex subgroups (W = 6.2) than the PR = 1 model (W = 2.1). Distribution differences across the disease subgroups remain fairly unchanged (W = 3.8 and W = 4.1). Across the 1000 sampling runs, the medians and the interquartile ranges of the Wasserstein distances were 2.1 [1.9, 2.3] for the sex subgroups and 4.1 [3.6, 4.5] for the disease subgroups in the balanced trained model, and 6.2 [5.7, 6.7] and 3.8 [3.4, 4.2], respectively, in the imbalanced trained model.

## Discussion

In this study, we addressed a critical gap in the existing literature by investigating the presence of sex bias in deep learning models for PAT imaging, specifically within the context of PAD diagnosis. Our results showed that sex can be predicted solely from PAT images, suggesting that neural networks may engage in shortcut learning, which could lead to performance disparities in diagnoses between sexes. Additionally, we are the first to explore shared feature representations of sex and PAD as a potential reason for shortcut learning in PAT.

We demonstrated that CNNs can effectively classify sex from PAT images of calf muscles, which reinforces that PAT images contain sufficient information for neural networks to distinguish between sexes. It raises awareness that models trained on PAT data may inadvertently learn and utilize sex-related characteristics potentially leading to biased predictions. We could show that the sex classifier generalizes despite the limited sample size to an out-of-distribution (OOD) dataset partly consisting of body sites that were not in the training dataset. Notably, classification on calf images surpassed in-distribution performance, possibly due to a younger cohort or acquisition protocol variations leading to higher image quality.

*Identification of sex bias and its impact on PAD diagnosis models*

We demonstrated that models trained on datasets with imbalanced sex-specific PR show significant performance degradation (up to 0.21 AUROC drop) when tested on balanced datasets. This indicates that these models are sensitive to shifts in sex-disease prevalence between training and deployment environments. As a result, the models do not generalize well to populations with different sex distributions, leading to decreased performance in real-world settings. In contrast, models trained on datasets with balanced PR provide robust performances independent of sex-disease prevalence shifts, highlighting the importance of dataset composition in mitigating shortcut behavior.

We could also show that the underdiagnosis disparity between sexes increases as the sex-specific PR in the training data increases. Models trained on data with increasing sex-specific PR were more likely to underdiagnose the sex that was underrepresented among the diseased individuals in the training data. This might occur because the model predominantly learns disease features from the overrepresented sex (males in this case), leading to a lack of generalization to the underrepresented sex (females). Note that the higher underdiagnosis disparity for PR = 5 compared to PR = $\infty$ is most likely attributable to our rather small test set with a sample size of 40.

*Evidence of shortcut learning through shared feature representations*

We showed that there is a considerable similarity between the neural network representations for sex and PAD features, enabling effective transfer learning between these tasks. The first two principal components of the model trained on a dataset with PR = $\infty$ exhibited a more pronounced disparity across the sex subgroups compared to the model trained on a dataset with PR = 1 (cf. Fig. 7), indicating a stronger sex-biased encoding for models trained on data with high PR. In Fig. 6, performance drops from top right to bottom left (and vice versa), indicating that the encoders do not extract purely task-agnostic features. If they were entirely task-agnostic, we would expect no performance loss when retraining the heads for the alternate task. Nonetheless, the positive transfer learning result confirms that some features remain useful. The precise ratio of task-agnostic versus task-specific but transferable features remains challenging to determine.

The sex of a patient is routinely considered in certain medical diagnoses [25]. While sex is an important and legitimate factor in most diagnostic contexts, the concern arises when models develop an overreliance on sex-derived features instead of learning direct markers of disease. In this study, we simulated scenarios where training data exhibited unrepresentative sex-specific prevalence ratios (PR > 1) and provided evidence that such data distributions promote shortcut learning. It is worth noting that this does not challenge the diagnostic relevance of sex in PAD, but rather highlights how classifiers may prioritize sex-related features over physiological disease markers, leading to biased and non-generalizable predictions. Such biases can compromise model robustness and fairness, particularly when sex-specific prevalence shifts between training and deployment populations. Future research should explore the effectiveness of explicitly incorporating sex as an auxiliary feature in a controlled and interpretable manner, rather than allowing models to infer sex implicitly in a way that may exacerbate shortcut learning.

Compared to other studies in the field of PAT, as outlined in a recently published review paper [26], our sample size lies within the 90th percentile regarding the number of subjects. Investigations on even larger, preferably multi-center studies should nevertheless be subject to future work. Furthermore, while we demonstrate sex bias, other confounding factors (e.g., age, comorbidities) were not explicitly controlled for, which may also influence model performance. Further, this study focuses on PAD diagnosis, and while the results are likely relevant to other applications, further research is needed to assess the impact of sex bias in different clinical settings. To improve the performance of the PAD classifier (AUROC: 0.79, 95% CI: 0.60$-$ 0.94), we tested several optimization strategies, including gradual unfreezing of the EfficientNetV2 backbone, however none yielded meaningful gains. These efforts suggest that dataset limitations, rather than model architecture, constrain performance. While a larger dataset could enhance overall performance, it remains unclear whether a stronger model would still rely on sex-based shortcuts. In addition, although we identified sex bias in the models, we did not explore techniques for mitigating these biases such as using a weighted loss which could further improve performance, especially in settings with PR$\ne$1 and thus attempting to mitigate the reliance on sex-based shortcuts. However, we opted not to implement these techniques for reducing the risk of shortcut learning in this work to provide an unfiltered view of the risks caused by sex-based shortcuts. Future work should investigate methods for bias reduction, such as data augmentation or fairness-aware learning algorithms.

In conclusion, this study highlights the critical issue of sex bias in deep learning models for PAT-based PAD diagnosis, arising from models’ unintended and spurious reliance on sex-related features due to shortcut learning. Our findings underscore the importance of carefully designing training datasets-particularly taking into account imbalances in sex-specific PR-to prevent shortcut learning and ensure the development of fair and reliable artificial intelligence models for medical imaging.

## Supplementary information

Additional details on the data and model training can be found in the material.

## Supplementary Information

Below is the link to the electronic supplementary material.

Supplementary file 1 (pdf 226 KB)
