# Supplementary material for: Shortcut learning leads to sex bias in deep learning models for photoacoustic tomography
Source: Int J Comput Assist Radiol Surg. 2025 May 9;20(7):1325–33. doi: 10.1007/s11548-025-03370-9 (PMC12226672; doi:10.1007/s11548-025-03370-9)
Supplement: Supplementary file 1 — (pdf 226 KB) [file 11548_2025_3370_MOESM1_ESM.pdf]

Supplementary Material for: Shortcut Learning Leads to Sex Bias in Deep Learning Models for Photoacoustic Tomography

Marcel Knopp[1,2*†], Christoph J. Bender[1,3*†], Niklas Holzwarth[1,2], Yi Li[4], Julius Kempf[4], Milenko Caranovic[4], Ferdinand Knieling[5], Werner Lang[4], Ulrich Rother[4‡], Alexander Seitel[1,6‡], Lena Maier-Hein[1,2,3,6*‡], Kris K. Dreher[1,7‡]

[1*]Division of Intelligent Medical Systems (IMSY), German Cancer Research Center (DKFZ), Heidelberg, Germany.
[2]Faculty of Mathematics and Computer Science, Heidelberg University, Heidelberg, Germany.
[3]Medical Faculty, Heidelberg University, Heidelberg, Germany.
[4]Department of Vascular Surgery, University Hospital Erlangen, Friedrich-Alexander-Universität Erlangen-Nürnberg (FAU), Erlangen, Germany.
[5]Department of Pediatrics and Adolescent Medicine, University Hospital Erlangen, FAU, Erlangen, Germany.
[6]National Center for Tumor Diseases (NCT), NCT Heidelberg, a partnership between DKFZ and University Hospital Heidelberg, Heidelberg, Germany.
[7]Faculty of Physics and Astronomy, Heidelberg University, Heidelberg, Germany.

*Corresponding author(s). E-mail(s): {marcel.knopp, christophjulien.bender, l.maier-hein}@dkfz-heidelberg.de;
†These authors contributed equally and share first authorship.
‡These authors shared equal leadership in this work.

S1 Reproducibility

This section describes the methods and resources used to ensure reproducibility of our experiments, detailing the model hyperparameters and training details (S1.1), the computational infrastructure (S1.2).

S1.1 Model and training

The model hyperparameters and training configurations used in our experiments are presented in Table S1.

Table S1: Hyperparameters used in all experiments

**Config**	**Value**
Architecture	EfficientNetV2_B0
Optimizer	AdamW<br>- lr: 1×10^{-4}<br>- β_1: 0.9<br>- β_2: 0.999<br>- weight decay: 1×10^{-5}
Max Epochs	100
Early stopping	Monitor: validation loss<br>patience: 10 epochs
Best epoch selection	Based on validation loss
Initialization	ImageNet-pretrained weights<br>(imported by timm [1])
Augmentation	RandomResizedCrop(224)<br>RandomRotation(15°)<br>RandomHorizontalFlip
Batch size	8
Ensemble size	10 models

Training was done with the hardware stated in Table S2, with an average runtime of 3.16 s per 10 epochs. All ensembles can be trained and evaluated using less than 6 GB VRAM.

S1.2 Computing infrastructure

To carry out our computational experiments, we used the following hardware (cf. Table S2) and software setup (cf. Table S3):

Table S2: Hardware resources used

Component	Specification
GPU	NVIDIA RTX 3090 with 24 GB VRAM
CPU	AMD® Ryzen 9 5900x 12-core processor × 24
RAM	64 GB DDR4 memory

Table S3: Software frameworks and versions used

Component	Specification	
OS	Ubuntu 22.04.5 LTS	
CUDA	11.8.89	
Python	3.9.18	
Frameworks	PyTorch [2, 3]	2.0.1+cu118
	Torchvision [4]	0.15.2+cu118
	Timm [1]	1.0.9
	Scikit-learn [5]	1.3.0
	Pandas [6, 7]	2.0.3
	Numpy [8]	1.25.2

S2 Datasets

This section provides further details of the datasets used in our study, specifically the peripheral artery disease (PAD) dataset (S2.1) and the datasets of the healthy volunteers (S2.2). For each of them, we show the distribution of the sex-disease strata (S2.1.1 and S2.2.1), and the processing steps implemented to prepare the data for analysis (S2.1.2 and S2.2.2).

S2.1 Peripheral artery disease (PAD) dataset

This subsection offers additional insights into the PAD dataset. The data were collected as part of two clinical studies at the Department of Vascular Surgery, University Hospital Erlangen, Germany under the study IDs NCT05373927 and NCT05773534. In these studies, the calves of the participants were measured before and after an exercise or our analysis, we exclusively used the 2D images captured before exercise to exclude other exercise-related confounding factors and to eliminate differences between studies.

S2.1.1 Data distribution

We divided the 147 samples into three distinct pools: training, validation, and test data pools. To prevent any inadvertent leakage of data, the pools were kept constant throughout the course of the study. The data pools were then utilized, as described

in the main paper, to create training and validation sets with varying sex-specific prevalence ratios (PR) (cf. Table 1 in the main paper).

Table S4: Sex-disease strata distributions within the data pools

Pool	Female Healthy	Female Diseased	Male Healthy	Male Diseased	Total
**Training**	24	16	20	26	86
**Validation**	6	4	4	7	21
**Test**	12	7	9	12	40
**Overall Data**	42	27	33	45	147

S2.1.2 Data processing

Data reconstruction and preprocessing were performed using the open-source toolkit for simulation and image processing for photonics and acoustics (SIMPA) [9]. Time series data acquired with the CE-certified MSOT Acuity Echo (iThera Medical GmbH, Munich, Germany) underwent bandpass filtering using a Tukey window with an alpha value of 0.5, a high-pass cutoff of 50 kHz, and a low-pass cutoff of 10 MHz. Energy laser correction was applied for each wavelength. Temporal averaging was applied using eight frames for each wavelength, enhancing the signal-to-noise ratio. We corrected for time shifts in the signals of sensors showing an early response to remove artifacts. The image data were reconstructed using the Delay and Sum Reconstruction algorithm with a spacing of 0.1 mm. Each multispectral image consists of three channels corresponding to wavelengths of 760 nm, 800 nm, and 850 nm. The images were cropped to a dimension of 400×256 pixels and z-score normalized using the channel-wise mean and standard deviation calculated from the training dataset.

S2.2 Out-of-distribution (OOD) datasets of healthy volunteers

This subsection offers additional insights into the datasets of the healthy volunteers. The healthy volunteer dataset contains PAT images that were acquired at the calf, the forearm, and the neck. For each of these body sites images were taken at up to three distinct locations both on the left and right side of the body yielding N=525 images in total. The dataset was acquired in extension to a previously published study where the acquisition procedure and other specifics can be read [10].

S2.2.1 Data distribution

To assess the generalizability of our sex classifier, we evaluated it on three out-of-distribution (OOD) datasets consisting of photoacoustic tomography (PAT) images of human calves, forearms, and necks from healthy volunteers. These datasets were also acquired using an MSOT Acuity Echo (iThera Medical GmbH, Munich, Germany) and included up to three scans per subject, per side (left and right), and per body region

(calf, forearm, neck), with the total counts summarized in Table S5. The same stratified bootstrapping ($n_{iter} = 1000$) was applied to generate 95 % confidence intervals (CIs) for the reported area under the receiver operating characteristic curve (AUROC) scores in Figure 3. For this, we used a sex-balanced sample count distribution aligned with PR = 1, resulting in 66 images per sex.

Table S5: Sex distributions in healthy volunteer data (OOD test sets)

Region	Total Images	Unique Patients	Female	Male
**Calf**	176	30	12 (70 images)	18 (106 images)
**Forearm**	174	29	11 (66 images)	18 (108 images)
**Neck**	175	30	12 (72 images)	18 (103 images)

S2.2.2 Data processing

Data reconstruction and preprocessing were performed as for the PAD dataset (described in S2.1.2), with two key modifications. Due to the limited number of frames in some measurements, temporal averaging was performed using seven frames per wavelength instead of eight. To mitigate complex parasitic noise [11], while preserving the tissue signal, we employed a singular value decomposition (SVD)-based noise reduction technique [12] on the time series data from the outer sensor bands (sensors 1–32 and 225–256)

References

[1] Wightman, R.: PyTorch Image Models. GitHub (2019). https://doi.org/10.5281/zenodo.4414861

[2] Paszke, A., Gross, S., Massa, F., Lerer, A., Bradbury, J., Chanan, G., Killeen, T., Lin, Z., Gimelshein, N., Antiga, L., Desmaison, A., Kopf, A., Yang, E., DeVito, Z., Raison, M., Tejani, A., Chilamkurthy, S., Steiner, B., Fang, L., Bai, J., Chintala, S.: Pytorch: An imperative style, high-performance deep learning library. In: Wallach, H., Larochelle, H., Beygelzimer, A., d'Alché-Buc, F., Fox, E., Garnett, R. (eds.) Advances in Neural Information Processing Systems 32, pp. 8024–8035. Curran Associates, Inc., ??? (2019). http://papers.neurips.cc/paper/9015-pytorch-an-imperative-style-high-performance-deep-learning-library.pdf

[3] Ansel, J., Yang, E., He, H., Gimelshein, N., Jain, A., Voznesensky, M., Bao, B., Bell, P., Berard, D., Burovski, E., Chauhan, G., Chourdia, A., Constable, W., Desmaison, A., DeVito, Z., Ellison, E., Feng, W., Gong, J., Gschwind, M., Hirsh, B., Huang, S., Kalambarkar, K., Kirsch, L., Lazos, M., Lezcano, M., Liang, Y., Liang, J., Lu, Y., Luk, C., Maher, B., Pan, Y., Puhrsch, C., Reso, M., Saroufim, M., Siraichi, M.Y., Suk, H., Suo, M., Tillet, P., Wang, E., Wang, X., Wen, W.,

Zhang, S., Zhao, X., Zhou, K., Zou, R., Mathews, A., Chanan, G., Wu, P., Chintala, S.: Pytorch 2: Faster machine learning through dynamic python bytecode transformation and graph compilation. In: 29th ACM International Conference on Architectural Support for Programming Languages and Operating Systems, Volume 2 (ASPLOS '24). ACM, ??? (2024). https://doi.org/10.1145/3620665.3640366 . https://pytorch.org/assets/pytorch2-2.pdf

[4] TorchVision maintainers, TorchVision contributors: TorchVision: PyTorch's Computer Vision Library

[5] Pedregosa, F., Varoquaux, G., Gramfort, A., Michel, V., Thirion, B., Grisel, O., Blondel, M., Prettenhofer, P., Weiss, R., Dubourg, V., Vanderplas, J., Passos, A., Cournapeau, D., Brucher, M., Perrot, M., Duchesnay, E.: Scikit-learn: Machine learning in Python. Journal of Machine Learning Research **12**, 2825–2830 (2011)

[6] team, T.: pandas-dev/pandas: Pandas (2020). https://doi.org/10.5281/zenodo.3509134 . https://pandas.pydata.org/

[7] McKinney, W.: Data structures for statistical computing in python. In: Walt, S., Millman, J. (eds.) Proceedings of the 9th Python in Science Conference, Austin, TX, US, pp. 56–61 (2010). https://doi.org/10.25080/Majora-92bf1922-00a

[8] Harris, C.R., Millman, K.J., Walt, S.J., Gommers, R., Virtanen, P., Cournapeau, D., Wieser, E., Taylor, J., Berg, S., Smith, N.J., Kern, R., Picus, M., Hoyer, S., Kerkwijk, M.H., Brett, M., Haldane, A., Río, J., Wiebe, M., Peterson, P., Gérard-Marchant, P., Sheppard, K., Reddy, T., Weckesser, W., Abbasi, H., Gohlke, C., Oliphant, T.E.: Array programming with NumPy. Nature **585**, 357–362 (2020) https://doi.org/10.1038/s41586-020-2649-2

[9] Gröhl, J., Dreher, K.K., Schellenberg, M., Rix, T., Holzwarth, N., Vieten, P., Ayala, L., Bohndiek, S.E., Seitel, A., Maier-Hein, L.: SIMPA: an open-source toolkit for simulation and image processing for photonics and acoustics. Journal of Biomedical Optics **27**(8), 083010 (2022) https://doi.org/10.1117/1.JBO.27.8.083010

[10] Schellenberg, M., Dreher, K.K., Holzwarth, N., Isensee, F., Reinke, A., Schreck, N., Seitel, A., Tizabi, M.D., Maier-Hein, L., Gröhl, J.: Semantic segmentation of multispectral photoacoustic images using deep learning. Photoacoustics **26** (2022) https://doi.org/10.1016/j.pacs.2022.100341 . Accessed 2025-01-09

[11] Dehner, C., Olefir, I., Chowdhury, K.B., Jüstel, D., Ntziachristos, V.: Deep-Learning-Based Electrical Noise Removal Enables High Spectral Optoacoustic Contrast in Deep Tissue. IEEE Transactions on Medical Imaging **41**(11), 3182–3193 (2022) https://doi.org/10.1109/TMI.2022.3180115 . Conference Name: IEEE Transactions on Medical Imaging. Accessed 2025-01-10

[12] Hill, E.R., Xia, W., Clarkson, M.J., Desjardins, A.E.: Identification and removal of

laser-induced noise in photoacoustic imaging using singular value decomposition. Biomedical Optics Express **8**(1), 68–77 (2017) https://doi.org/10.1364/BOE.8.000068 . Publisher: Optica Publishing Group. Accessed 2025-01-10
